# What stakeholders think: perceptions of perinatal depression and screening in China’s primary care system

**DOI:** 10.1186/s12884-020-03473-y

**Published:** 2021-01-06

**Authors:** Shahirose Sadrudin Premji, Keith S. Dobson, Anupa Prashad, Shelby Yamamoto, Fangbiao Tao, Beibei Zhu, Xiaoyan Wu, Mengjuan Lu, Shanshan Shao

**Affiliations:** 1 School of Nursing, Faculty of Health, York University, HNES 313, 4700 Keele Street, Toronto, Ontario M3J 1P3 Canada; 2 Department of Psychology, University of Calgary, Administration 235A, 2500 University Drive NW, Calgary, Alberta T2N 1N4 Canada; 3 School of Public Health, University of Alberta, 3-300 Edmonton Clinic Health Academy, 11405-87 Ave, Edmonton, Alberta, T6G 1C9 Canada; 4 Department of Maternal, Child and Adolescent Health, School of Public Health, Anhui Medical University, 81 Meishan Road, Hefei, 230032 People’s Republic of China; 5 Anhui Provincial Key Laboratory of Population Health & Aristogenics, Hefei, People’s Republic of China

**Keywords:** Implementation science, Qualitative, Perinatal depression, CFIR

## Abstract

**Background:**

Mental health in China is a significant issue, and perinatal depression has been recognized as a concern, as it may affect pregnancy outcomes. There are growing calls to address China’s mental health system capacity issues, especially among vulnerable groups such as pregnant women due to gaps in healthcare services and inadequate access to resources and support. In response to these demands, a perinatal depression screening and management (PDSM) program was proposed. This exploratory case study identified strategies for successful implementation of the proposed PDSM intervention, informed by the Consolidated Framework for Implementation Research (CFIR) framework, in Ma’anshan city, Anhui province.

**Methods:**

This qualitative study included four focus group discussions and two in-depth individual interviews with participants using a semi-structured interview guide. Topics examined included acceptance, utility, and readiness for a PDSM program. Participants included perinatal women and their families, policymakers, and healthcare providers. Interviews were transcribed verbatim, coded, and analyzed for emergent themes.

**Results:**

The analysis revealed several promising factors for the implementation of the PDSM program including: utilization of an internet-based platform, generation of perceived value among health leadership and decision-makers, and the simplification of the screening and intervention components. Acceptance of the pre-implementation plan was dependent on issues such as the timing and frequency of screening, ensuring high standards of quality of care, and consideration of cultural values in the intervention design. Potential challenges included perceived barriers to the implementation plan among stakeholders, a lack of trained human health resources, and poor integration between maternal and mental health services. In addition, participants expressed concern that perinatal women might not value the PDSM program due to stigma and limited understanding of maternal mental health issues.

**Conclusion:**

Our analysis suggests several factors to support the successful implementation of a perinatal depression screening program, guidelines for successful uptake, and the potential use of internet-based cognitive behavioral therapy. PDSM is a complex process; however, it can be successfully navigated with evidence-informed approaches to the issues presented to ensure that the PDSM is feasible, effective, successful, and sustainable, and that it also improves maternal health and wellbeing, and that of their families.

**Supplementary Information:**

The online version contains supplementary material available at 10.1186/s12884-020-03473-y.

## Background

Estimates of the rates of antenatal depression in China have ranged from 6 to 28%, while depression rates in the first postpartum year have ranged from 10 to 28% [1–4]. Antenatal depression increases rates of health care utilization, such as non-scheduled antenatal care or emergency care for pregnancy-related emergencies [5]. Untreated antenatal depression can have harmful consequences for both mother (e.g., suicide, disease mortality, postpartum depression) and baby (e.g., preterm birth, neurodevelopmental problems) [6–11].

Anhui province, located in Southeast China with an economy (low gross domestic product) based on farming and less developed rural areas, has prenatal care services in every township [12]. Anhui province was found to have the highest prevalence of perinatal depression (33%) in a meta-analysis including 95 studies from 23 regions in Mainland China [13]. Rates of screening, diagnosis, and availability of effective treatments to reduce perinatal depression are low worldwide, but more so in China and Anhui province in particular [13, 14]. Chinese women’s mental health needs, especially those in underdeveloped regions, are unmet given lack of investment in mental health, unequal geographical distribution of mental health services, and the lack of trained or specialized healthcare providers [13–15].

We planned and developed a perinatal depression screening and management (PDSM) program, with the intent to integrate the program within primary care and to utilize internet-based cognitive behavioral therapy (iCBT) to effectively deal with system capacity issues in Anhui province, China [16–18]. Evidence suggests that integrating the PDSM within primary care will lead to better follow-up and initiation of therapy, given improved processes for managing depression; hence, improved maternal/child health outcomes [19]. Internet-based cognitive behavioral therapy (iCBT) has been shown to reduce depressive symptoms (effect size 0.42 to 0.65) in 26 randomized controlled trials, without imposing disproportionate demands on specialized healthcare providers, or increasing costs or wait times for care [16, 17, 20].

Although perinatal depression screening and iCBT are effective tools to increase mental healthcare accessibility and reduce the risk of perinatal depression, evidence-practice gaps are apparent worldwide, including in China [13, 14]. Stakeholders may unintentionally contribute to these evidence-practice gaps in unpredictable or harmful ways [21]. For instance, community healthcare workers in Guangzhou, China demonstrated limited awareness of perinatal depression [22]. Evidence suggests that these providers may have even inadvertently perpetuated stigma towards mental illness or mental health patients, by failing to openly discuss a mental health diagnosis [22]. Uptake and adherence to digital interventions vary based on recipient’s access to the internet, appeal of the intervention, willingness to participate, and perspectives related to the benefits of the digital intervention [23]. Understanding provider and recipient’s readiness for perinatal depression screening and management can assist with the successful integration of the PDSM program including uptake of iCBT within primary care [24]. A PDSM program that combines interventions for mothers and providers, and addresses practice barriers, has been shown to be more efficacious in detecting, referring and treating depression compared to screening alone [24].

Both the American College of Obstetricians and Gynecologists (ACOG) [25] and the Australian National Perinatal Depression Initiative (NPDI) [26] assert that pregnant women should be screened at least once using a standardized, validated measurement tool. Antenatal depression rates differ across timing of pregnancy [27]. Among women in six counties/districts in six Chinese provinces, antenatal depression rates were 14% in the first trimester, 13% in the second trimester, and 11% in the third trimester [28]. These rates of antenatal depression were determined using the Hospital Anxiety and Depression Scale (HADS); though a systematic review reports many advantages of using the Edinburgh Postnatal Depression Scale (EPDS) in low resource settings (e.g., moderate to high accuracy, high sensitivity, and high specificity) [29]. Similarly, the Chinese version of the EPDS is valid and reliable for detecting antenatal depression [30], as well as postpartum depression [31]. It is prudent, however, to consider policymakers’ perspectives when adapting guidelines, as policymakers hold invaluable knowledge regarding context (e.g., different prevalence of depression across timing of pregnancy, countries, and hospital) [14, 32] and making changes in practice [29].

The aim of this pilot study was to engage multiple stakeholders (i.e., healthcare providers, pregnant and postpartum women and their families, and policymakers), to explore the acceptance, utility and readiness for a PDSM program in Anhui Province, where perinatal depression has become a public health concern. The goal was to assess the integration of perinatal depression screening and iCBT within a flexible framework that would consider context, ensure that mental healthcare is responsive to women’s needs, and promote implementation outcomes of acceptance, reach, scope, and scale. Identification of implementation strategies, in this context, was expected to improve uptake and knowledge for eventual program expansion. In addition, this case study also contributes to the body of implementation science research as it is guided by the five domains of the Consolidated Framework for Implementation Research (CFIR) framework to promote implementation of the PDSM and iCBT and its effectiveness [21, 33]. CFIR considers: (a) **intervention** including determining aspects of the PDSM and iCBT program that are core and should remain unchanged (e.g., screening, iCBT), and areas that can be modified (e.g., tools, frequency); (b) **process** of implementing PDS and iCBT within primary care including engaging healthcare providers in adapting PDSM and iCBT, and providing adequate training and support; (c) **individuals involved** such as healthcare providers’ and perinatal women’s knowledge, acceptance, and perceived strengths and challenges that need to be addressed, and strategies that will promote adherence to the iCBT intervention; (d) **inner setting** such as considering the availability, qualification and reputation of healthcare workers, ensuring collaboration between maternal and newborn care practitioners and department of psychiatry, and engaging leadership; and (e) **outer setting** such as addressing funding for the PDSM and iCBT program (i.e., ensuring affordability for perinatal women). The CFIR enables a better understanding of the complexities of implementation and implementation effectiveness of the PDSM and iCBT [21]. Moreover, it can provide direction for data collection and analysis during the implementation that facilitates rapid-cycle improvements and determine “what works where and why across multiple contexts” [21] (p. 2).

## Methods

The pilot study conducted at the Ma’anshan Maternal and Child Healthcare Center, Ma’anshan city, Anhui province, China involved three semi-structured, in-depth interviews and three focus groups (see supplemental files for interview guides) that each comprised 3 to 7 participants. Participants were purposively-selected and included: (a) three policymakers (age range 48–52 years, two female), all of whom held university degrees and worked in executive roles related to maternal and child health care in Ma’anshan; (b) six healthcare staff (age range 32–42 years, five female), all of whom had at least an undergraduate university degree and were employed in either mental or maternal health at the primary care perinatal facility; (c) group of six pregnant/postpartum women (age range 24–42 years) who sought services at the maternal and child health care in Ma’anshan that included only screening and no intervention for women with depressive symptoms, five of whom had undergraduate university degrees, and four of whom were employed in roles that included hotel staff, law clerk, nurse and teacher; and (d) group of seven family members (age range 25–55 years; four male) who accompanied the women for seeking maternal and child health care. Engaging diverse stakeholders enabled greater confidence in interpretations of findings at multiple levels of the healthcare system.

Policymakers and healthcare providers including physicians, nurses and midwifes employed by the China Maternal and Child Healthcare System were sent an invitation letter from the study team. Pregnant/postpartum women and their accompanying family members (husband or mothers) were invited to participate in the study through an invitation letter shared by their healthcare provider. The data was collected in August 2018 by trained research team members with previous engagement in qualitative research. Institutional review board approval was obtained at Anhui Medical University, York University, University of Alberta, and the University of Calgary, and all participants completed the informed consent process.

The data and field notes were audio-recorded, transcribed verbatim in Chinese, translated to English, and triangulated by another research team member to ensure language equivalency. Two researchers independently reviewed English transcripts and highlighted significant statements, sentences, quotes or words that were categorized into clusters of meaning through coding data [34, 35]. Patterns were identified in the coded data to determine central themes and relationships across narratives. When there was lack of consensus about patterns, themes, or relationships, the researchers returned to the data to reconcile discrepancies. The study objective was to identify implementation strategies for the successful uptake of a PDSM program. Accordingly, codes were developed based on emerging themes that were relevant to determine feasibility, acceptance, and reach of the PDSM program. Additionally, this study examined the effects of contexts that could potentially enable or restrict utilization of the PDSM program.

## Results

**Fig. 1:**
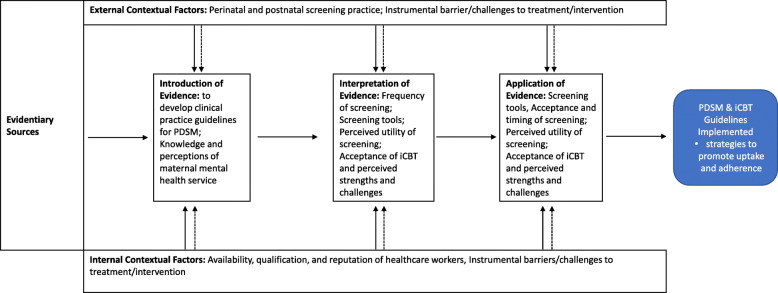
Themes and how they affect what constitutes evidence and how it may be utilized [36].

The interview data illuminated several themes that were relevant to perinatal screening and the proposed iCBT intervention. These themes were: Availability, Qualifications, and Reputation of Healthcare Workers, Acceptance and Timing of Screening, Frequency of Screening, Perceived Utility of Screening, Screening Practices, Screening Tools, Acceptance of iCBT, Knowledge and Perceptions of Maternal Mental Health Services, and Instrumental Barriers/Challenges to Treatment/Intervention. Each of these themes is discussed in turn below. Fig. 1 presents a conceptualization of how these themes affect what constitutes evidence, how evidence may be interpreted and applied, and the decision-making context (i.e., internal and external contextual factors) [36]. The inter-relationship is evident with themes re-appearing within evidence utilization and within contexts.

### Availability, qualifications, and reputation of healthcare workers

The data suggests an insufficiency of trained or specialized healthcare providers, who are qualified to provide psychosocial and mental health supports for women who screen positive for perinatal depression. For example, one participant said: “Professional personnel are actually lacking” (Healthcare provider #3). Another participant said: “In county level they lack talents badly. The clinics are saturated with patients. We do not have adequate mental health services in this area. Especially we do not have adequate professionals to intervene” (Policymaker #1).

In addition to concerns about the number of trained healthcare workers, participants also commented about the importance of qualifications: “The question of qualifications is important … It’s best to have widely recognized qualification[s]” (Healthcare provider #4). The importance of high standards for health workers was also emphasized and tied to qualifications: “It must be th[at] people under[go] three to five years medical training. The people [who] only have a psychological counselor’s certificate, we don’t want to hire.” A policymaker added that, “We would rather go without than having someone shoddy” (Policymaker #1). Another healthcare provider talked about the importance of licensure, and said, “I think as long as you’re a general professional, there is a professional license. You’re protecting yourself, including patients, as long as there is a license, it is okay” (Healthcare provider #2). Finally, according to another participant, healthcare providers with good credibility and qualifications were important for successful outcomes: “[It] may ensure that people believe in and recognize the treatment more” (Healthcare provider #4). Given the limited resources and the time required to build health worker capacity, iCBT was considered a suitable alternative. To address human resource shortages, participants emphasized the need for training: “I think we have the abilities to do that. We can enhance our training to complete this screening process” (Policymaker #2).

### Acceptance and timing of screening

Participants expressed varying degrees of acceptance for the management of depressive symptoms in perinatal women. One healthcare provider recalled that, “In 2012, postpartum screening was already being done” (Healthcare provider #3) whereas according to another provider screening during pregnancy “only started in 2015” (Healthcare provider #3). Focus group discussions with healthcare providers revealed that perinatal screening was widely implemented at the municipal level and had significant coverage. One policymaker described perinatal depression screening as a “routine healthcare task, covering at least 90% of the pregnancy population” (Policymaker #1).

Varied opinions emerged about the optimal frequency and timing of screening. For example, healthcare providers discussed perinatal screening at the municipal healthcare level and stated that screening “starts in early pregnancy” (Healthcare provider #1). They added that, “during the pregnancy period, if outpatient doctors found psychological problems, they direct her to the psychological clinic” (Healthcare provider #1). The healthcare provider explained, “If it is severe… That is to say, if she needs drug treatment, she will be treated at the upper level like in a mental health/ psychiatric center” (Healthcare provider #1). Healthcare providers noted that postpartum screening occurred twice: initially during a postpartum visit when the mother visited the clinic, and once again at the 42-day postpartum visit. Another healthcare provider commented: “When she comes to me for a medical examination in 42 days, I’ll give her another assessment” (Healthcare provider #2).

While some participants emphasized the importance of prenatal screening, others emphasized postpartum screening. For example, one healthcare provider said: “The highest proportion of those with perinatal depression are in [the] postpartum [period]” (Healthcare provider #4). Another healthcare provider added that, “42 days after childbirth [it] must be done once, because after childbirth, the family is completely chaotic, [childbirth] has a big psychological impact on the mother” (Healthcare provider #2). Participants also described stressors and lifestyle changes encountered in the postpartum period, which was why they suggested screening during that time. For example, one perinatal woman added, “It’s when you stay at home, especially when everyone else is at work. You are alone at home, just whimsical … nothing to do, it is easy to feel emotionally unwell” (Perinatal woman #2). Other women shared this sentiment and advocated post-partum screening: “You could screen within 28 days [of birth], and once again at two to three months, and then six months after [birth]” (Perinatal woman #2). Other suggestions included: “42 days post-partum for the screening” (Perinatal woman #4), “within half a year [after giving birth]” (Perinatal woman #6), and “once or twice in month six to one year [postpartum]” (Perinatal woman #6). Finally, a family member suggested another time for screening: “One more [screening] can be done before the end of maternity leave” (Family member #4).

### Frequency of screening

Some participants supported frequent screenings that exceeded the minimum standard, while others had unfavorable views about screening too much. For example, one policymaker stated that, “one time is not enough” and proposed, “I think it may be two to three times, the first screening should be at the time when she visits [the] perinatal clinic. The second screening should be carried out not long before labour, one more time on the 42nd day after delivery” (Policymaker #2).

Pregnant and postpartum women expressed less favorable views for multiple screenings, as time constraints and conflicting commitments were cited as barriers. A pregnant woman explained: “A longer [appointment] time isn’t reasonable. A lot of pregnant women still have jobs… and personal time matters. A screening might drag on to one hour, 1.5 hours, people probably wouldn’t want to do something like that” (Perinatal woman #3). Another woman described her conflicting commitments, saying, “Back then I wanted to go to a counseling center in Nanjing to consult…the result was that I was too busy to go. Two kids and I didn’t have time to go” (Perinatal woman #5). Other participants agreed that shorter screenings would be more acceptable. One woman stated, “A shorter time is alright” (Perinatal woman #3). A postpartum woman commented, “It shouldn’t be too long nor too much…Check-up time is long and you have to line up, energy-wise we already can’t keep up” (Perinatal woman #4). Participants were concerned about additional appointments resulting from a screening component. One woman stated, “I like to save time, don’t want to do extra examinations” (Perinatal woman #6). Family members and perinatal women suggested adding the screening component to routine pregnancy and postpartum appointments. One woman stated, “It’d be good to do it at the same time as the pregnancy exam” (Perinatal woman #5).

### Perceived utility of screening

Many pregnant and postpartum women did not value mental health screening. A pregnant woman explained, “During pregnancy, some women when they go to medical examinations… do not place importance on this aspect, the psychological aspect…some people may not necessarily do these assessments” (Perinatal woman #2). Women may have felt this way due to several factors ranging from stigma, limited knowledge about mental health, or perceptions that they were unlikely to be affected by depression. Indeed, one pregnant woman discussed stigma as a reason why she would choose not to access mental health services because, “if your family found out, they’d say you were crazy” (Perinatal woman #5). Another woman explained that screening might be overlooked because perinatal women typically only engage with mental health services after attempting to self-manage their own depressive symptoms or when they experience severe emotional distress. She stated, “If it was serious then I’d go to the doctor. If it wasn’t serious then I’d go to friends, family, or self-manage” (Perinatal woman #4).

### Perinatal and postnatal screening practices

Pregnant and postpartum women reported inconsistent experiences with perinatal screening practices. One pregnant woman recalled that she was screened twice: “When we had a medical examination at [Named] Hospital, there was a postpartum emotional evaluation. [They] asked us [about depression] during the pregnancy exam and then after the birth” (Perinatal woman #5). In contrast, another woman indicated she was screened only once: “I only got it [screening] postpartum” (Perinatal woman #2). However, family members indicated that sometimes screening never occurred: “No screening was done” (Family member #6).

One policymaker stated that screening for depression was not compulsory: “Screening is not yet treated as a task that must be completed, because the qualifications and technical levels of personnel in this area have yet to be improved” (Policymaker #2). Participants also stated that training health workers must precede the implementation of policies for mandatory screening. When discussing mandatory screening, a healthcare provider suggested that human resources was an important component: “To add people, this is the minimum requirement. Without more employees it can’t be done. Really can’t do it” (Healthcare provider #5).

Perceptions about the quality of existing perinatal mental health screening varied and was tied to issues such as communication. One pregnant woman shared that: “Communicating with the doctor…is pretty good” (Perinatal woman #6). Similarly, another pregnant woman commented that her physician was ‘responsible’ because they asked her pertinent questions: “My obstetrician is really responsible, every appointment they’d ask about my mood, diet, and life” (Perinatal woman #4). In contrast, some women expressed their dissatisfaction about poor communication. For example, one woman disclosed that her healthcare provider did not communicate adequately and rushed through the process: “They wouldn’t give you much time to talk. They wouldn’t, they’d be annoyed with you. It’s like they want you to go in, get out quickly. So that’s why I didn’t talk to them” (Perinatal woman #5). A postpartum woman shared a similar experience. “During check-ups they ask you some basic questions about which stage you’re at in the pregnancy what steps things like that. Regarding the psychological aspects—not much so far” (Perinatal woman #6).

### Screening tools

One healthcare provider described the screening tools used for perinatal and postnatal depression: “In the early stage, the depression self-rating scale and the anxiety self-rating scale were used. In the second trimester and the third trimester, Hamilton depression and Hamilton anxiety were used. Postpartum, the Edinburgh Postnatal Depression Scale was used” (Healthcare provider #3). Another policymaker remarked: “The Edinburgh Assessment Scale we use is very simple” (Policymaker #1), and further suggested that healthcare providers could integrate this assessment into their practice with minimal training. They commented further: “The screening and assessment is very simple, only need to know how to use the scale” (Policymaker #1).

There was a notable lack of familiarity with various screening tools among policymakers. One policymaker said: “I’m not familiar with the screening” (Policymaker #2). They also said that they used other ways to screen for depression: “Now we don’t have a specific tool to exam[ine] them. One way is a postpartum interview, and the other way is that her family members tell us the symptoms she has” (Policymaker #2). Another policymaker struggled to recall the screening tools: “What tools are used, I really don’t remember” (Policymaker #1).

### Acceptance of iCBT and perceived strengths and challenges

Internet-based technology was generally considered to be an acceptable intervention for perinatal depression after positive screening results. This appraisal was based on perceptions of proficient internet access and usage among Chinese women. Most participants confirmed that perinatal women had access to the Internet via their smartphones, which could be leveraged with proper apps and services. Accordingly, a policymaker indicated that iCBT looked promising: “I personally think it is ok, because now people’s ability to use Internet is very high…They can use internet proficiently” (Policymaker #3).

Participants also indicated familiarity with the WeChat platform, which is a widely used internet-based communication program used throughout China. When describing her WeChat usage, a postpartum woman confirmed its popularity: “On one hand I’m feeding the baby [but] on the other, I’m on WeChat… WeChat is very popular now” (Perinatal woman #4).

Online technologies were considered advantageous because they helped to “reduce the workload of the doctors in clinic” (Policymaker #1) and enabled flexible, convenient support, efficient administration, and easy follow-up. One healthcare provider pointed out that, “It can be done at home, which is quite convenient” (Healthcare provider #3). A family member agreed with that sentiment: “I think this method is definitely more convenient and practical, and can be done anytime, anywhere” (Family member #6).

It is important to note that iCBT acceptance was modulated by the severity of depression, as participants viewed online intervention to be less acceptable for severe depression. A family member stated, “I think if the depression is severe, this method [iCBT] is inappropriate” (Family member #6) and another family member suggested a “face-to-face treatment for severe depression patients” and “online self-service therapy and counseling for light depression patients” (Family member #3).

Participants presented several possible challenges for iCBT in China. First, virtual iCBT means a lack of direct communication. Participants highlighted the importance of in-person interactions and live communication versus virtual ones: “The Internet, it’s impersonal” (Healthcare provider #6). Another healthcare provider shared a similar view: “I think it might be better to directly communicate. I personally feel better face-to-face” (Healthcare provider #6). Secondly, follow-up with mothers could be a challenge using iCBT. Healthcare workers encourage women to schedule appointments and ensure follow-up during in-person interactions; however, with iCBT these interactions might be lost. Third, some participants cautioned about the variability in norms, values, and customs in different provinces and regions across China, and how iCBT would need to be customized to the needs of the people: “We may have to take into account the habits of the Chinese people, it has to be converted…We want to treat one province as a unit, because the people’s habits in one province differ from another” (Policymaker #2). Fourth, participants suggested that iCBT might be considered as a temporary solution compared to in-person psychosocial support. For example, one policymaker explained that it was currently the only available option: “In the absence of psychological doctors it can help, it can solve some of the problems, but certainly it is not the best method, presently there is no better choice available, so it is the best” (Policymaker #1). As a fifth concern, some participants expressed that would be difficult to establish the credibility and qualifications of a healthcare provider online versus in-person. Indeed, one postpartum woman raised this issue: “I don’t know if that doctor is legitimate” (Perinatal woman #4). The perceived qualifications of the professionals who provide iCBT also influenced acceptance of the proposed PDSM program. For example, one postpartum woman said: “If there’s [a] qualified and licensed online doctor, I can accept it” (Perinatal woman #5). Sixth, participants discussed the challenges with respect to the significant time investment required for training: “It takes several years to train intervention diagnostic personnel, this is the hurdle” (Policymaker #1). They warned that building the health workforce will require substantial time stating, “It is not possible to train enough psychologists within three or five years, even in ten years” (Policymaker #1). Finally, one participant commented that follow-up after perinatal depression screening and diagnosis was inadequate and that she never received the required treatment: “But it’s just a review, and then there’s nothing else… we recovered on our own” (Perinatal woman #1).

### Knowledge and perceptions of maternal mental health services

According to participants, both maternal and child health were goals that could be accomplished by prioritizing maternal mental health. This idea suggested readiness for the PDSM program. For instance, one policymaker explained, “It should be said that the degree of attention is very high, we are the one of the first batch of National Childhood Early Development pilot project bases… We attach great importance to mental healthcare, and [are] fully aware of its importance” (Policymaker #1). Another participant prioritized maternal and child health above communicable diseases such as tuberculosis (TB): “It’s more important than the TB issue” (Policymaker #2). Finally, one healthcare professional said that leaders are, “gradually paying attention” (Healthcare provider #5) to maternal depression and that leadership is vital for the effective implementation of PDSM.

### Instrumental barriers/challenges to treatment/intervention

One participant explained the importance of interdisciplinary collaboration: “We have to rely on specialists in psychiatry and neurology for diagnosis, and we may not be able to do it ourselves” (Policymaker #2). The same participant suggested that obstetrics and psychiatry should work jointly to screen and treat depression, drawing on their collective expertise to “establish a perinatal care Standard Operation Procedure” (Policymaker #2). The logistics of this collaboration was questioned, however, raising concerns about the complexity of this process: “How [should we] combine our maternal and Child Health Care Services with the Department of psychiatry? Should the doctors in the Department of Psychiatry provide training for our people or should they participate in our work directly?” (Policymaker #2).

A policymaker voiced concern about mother’s adherence to online psychosocial support: “Some pregnant women may not do as they are supposed to do online, and I worry that it won’t work or go awry” (Policymaker #2). A healthcare provider shared a similar concern about compliance: “I’m afraid that pregnant women don’t have the patience and will skip to the last module right away …That is a very practical problem” (Healthcare provider #3). It was also recognized that efficacy would likely be compromised by poor compliance. Reflecting on the long-term commitment required for treatment and the time it takes to see the benefits, participants commented: “I feel like the Chinese… have no patience. Everyone wants to do something with proven effects. For example, if we give treatment, we say that psychological counseling cannot have an effect with only one counseling session, and then less people return for a second session” (Healthcare provider #3). A healthcare provider explained, “The curative effect prompts you to come. I think so, or why else do people come? I’ll come if your treatment has an effect” (Healthcare provider #2). Women who screen positively for depression may be reluctant to seek treatment as explained: “Slight depression, she always thinks, “I’m fine, then I didn’t have much effect after I got counseling. I’m basically a normal person. If she has this kind of thinking, she may not go to treatment voluntarily” (Healthcare provider #2).

Although funding for screening is provided by the municipal government, there were mixed responses about who should pay for care. For example, one healthcare provider stated: “[In] our city [the] government is paying for it” (Healthcare provider #2). Another participant said: “For screening, you might be able to have the government pay the bill” (Healthcare provider #1). However, a healthcare provider explained that this was not the case, and the patient paid for it out of pocket: “For treatment, it may still be the patient who pays” (Healthcare provider #3). The reason for this variability was due to diagnosis and treatment: “When you refer the patient to be diagnosed at a rehabilitation facility with the Disabled Persons’ Association, those treatments are free” (Healthcare provider #4). The cost of screening also varied, depending on the stage of the pregnancy: “We charge 40 yuan for depression and anxiety together, 40 in the first trimester, 40 in the second trimester, and free after childbirth” (Healthcare provider #3).

## Discussion

The study examined various aspects of the acceptability and potential implementation of a perinatal depression screening and management (PDSM) program in China. Based on a series of interviews with mothers, family members, health care providers, and policymakers, the results indicate that a PDSM program would be readily accepted and successful for mild to moderate depression, but not severe depression. The success of the program would be contingent, however, on the availability of adequate and qualified human resources, and a detailed protocol to ensure follow-up. Further, a thorough understanding of the context (socio-cultural, health care system) is needed to guide the implementation of this complex intervention within China’s Maternal and Child Healthcare System. The results of this study also suggest that various factors associated with the CFIR domains – intervention, process, individuals involved, inner setting, and out setting – that would facilitate a PDSM program already exist in China.

### Intervention, process, and individuals involved

Perinatal women were also amenable to screening, provided that such screening is aligned with the other maternal health services they access. Participants emphasized the need to ensure quality measures, such as hiring staff with appropriate qualifications, and ensuring privacy and confidentiality. Despite these positive responses, some challenges may be encountered during the process of implementation. Health behaviors, knowledge, and cultural beliefs about mental health may contribute to the unwillingness of Chinese women to participate in the intervention, thereby limiting its “reach”. It will likely be important to utilize educational components, to shift values and beliefs about mental health for women and families in the perinatal period.

Perinatal women believe that an internet-based intervention is flexible and can accommodate their numerous commitments as caregivers. Initially, the researchers had considered using a program that was developed and tested in Australia, entitled MoodGYM, but difficulties with server (slow speed) in the end precluded this option. Women had indicated acceptance concerning the use of the WeChat platform and were adept at using this platform. Consequently, an internet-based program based on principles of CBT but used the WeChat platform (entitled *Mom’s Good Mood)* was developed and implemented. It is important to establish both feasibility and acceptance of iCBT platform. Technical feasibility considerations include system performance and ease of use of technology as this can impact initiation and/or continued use of iCBT.

### Inner and outer settings

The urbanized location and setting of a particular healthcare organization and how it is networked with other external organizations are important criteria for the success of a PDSM program. Poor communication and unclear pathways of referral between maternal and mental health services would constitute significant barriers to the implementation of our PDSM program. It may be possible to overcome these obstacles by establishing a means of tracking referrals, introducing standard operating procedures, and transparent information sharing.

Identifying mothers’ needs and resource issues and addressing the barriers and facilitators to meet these needs and issues are imperative. The healthcare providers and policymakers identified challenges pertaining to health system and infrastructure challenges but did not readily identify the concerns expressed by the pregnant and postpartum women, such as time constraints and conflicting caregiving responsibilities. Of all the stakeholder groups, perinatal women conveyed the most reluctance towards the proposed intervention. It is critical for planners to avoid a top-down design, but instead include women’s voices in the design of screening and treatment, to maximize the acceptance of the program to its beneficiaries.

Internal organizational design and leadership capacity is a key construct in ‘readiness for implementation’. This construct entails components such as leadership engagement and available resources. Leadership engagement will facilitate the implementation of PDSM. The relevant leadership, health care providers and policymakers were receptive towards a prospective PDSM program, for the betterment of maternal mental healthcare management. In contrast, there was near unanimity about the need for additional staff and human resource support. Considerable work remains prior to implementation, and most poignantly to build a pool of trained health workers capable of treating women who screen positively for perinatal depression.

### Limitations

There were three primary limitations to this study. Interviews were conducted in Chinese and there was the possibility that language and the intended meanings may have been lost in translation. Efforts were made, however, to minimize this risk through triangulation and member-checking. Second, this study adopted a qualitative approach. Although qualitative data provides rich, detailed information, a mixed method approach could be adopted in the future to assess the key questions posed in this study. Such a methodology would enable further triangulation of the current results. Finally, this phase of the study was undertaken in Anhui province which is a relatively underdeveloped region in China. Hence, the extent to which these findings can be generalized to and across other regions in China may be limited.

## Conclusions

Several conclusions are possible for PDSM [37] in the context of China. Policymakers and healthcare providers discussed their ideas about the timing and frequency of screening and screening tools but did not explicitly reference evidence-based guidelines. There was a lack of certainty among health leadership with regards to the screening tools used, which implicates inconsistent use of evidence across contexts. Resource constraints, particularly health personnel, may limit the uptake of screening guidelines. Concerns also emerged among health leaders about the health system’s capacity to meet the extra demand for follow-up and treatment that would result from organized screening. Thus, although the literature suggests a strong need for mental health assessment and care in the perinatal period [14, 38–40], and that patients may receive significant benefit from on-line and other treatments [16, 41–43], there are considerable challenges to overcome in the delivery of these services in China [21, 44].

## Supplementary Information

**Additional file 1.** Interview Guide for Families. Interview Guide for Healthcare Provider. Interview Guide for Women. Interview Guide for Policymaker.

## Data Availability

The datasets generated and/or analysed during the current study are not publicly available due to reasons of privacy and confidentiality, however, de-identified data may be available from the corresponding author on reasonable request.

## Abbreviations

PDSM: perinatal depression screening and management
iCBT: internet-based cognitive behavioral therapy
TB: tuberculosis
CFIR: Consolidated Framework for Implementation Research
