# Supplementary material for: What stakeholders think: perceptions of perinatal depression and screening in China’s primary care system
Source: BMC Pregnancy Childbirth. 2021 Jan 6;21:15. doi: 10.1186/s12884-020-03473-y (PMC7789622; doi:10.1186/s12884-020-03473-y)
Supplement: Supplementary file 1 — Additional file 1. Interview Guide for Families. Interview Guide for Healthcare Provider. Interview Guide for Women. Interview Guide for Policymaker. [file 12884_2020_3473_MOESM1_ESM.zip › BMC Premji et al File 3_Guide FGD_WomenR4.docx]

**Interview Guide for Women**

Review Information Sheet and Consent Form and secure signatures.

Thank you for agreeing to participate in this interview and having this interview recorded. As explained, the interview will take approximately 60-90 minutes and will take the format of question and answers. If the question involves state secrets, you can also refuse to answer. The more detailed your answers to relevant questions, the better. At the end you will be given an opportunity to share anything that else that we have not covered. Thank you for your cooperation. Do you have any questions? If not, we are going to start the interview.

Before we begin, I am going to start recording and request that you indicate you give your permission to record this interview. You do not need to state your name or any other details that may identify you. Thank you!

**Section A. Universal Screening**

**Emotional distress such as depression affects many Chinese during pregnant and after the birth of their baby. Currently there is fair to moderate evidence that universal screening for depression should be integrated into primary care.**

1. Does the healthcare system currently give importance to women’s emotional wellbeing during pregnancy and/or up to one year after birth for depression?
2. What are your thoughts about screening all women during pregnancy and up to one year after birth in the primary healthcare system?
   1. Do you think women, pregnant or after birth, would be interested in or accept free screening for depression? Please explain. (Remind them to consider social discrimination, privacy disclosure, expense issues, time issues, etc.).
   2. Would you be interested in or accept free screening while you are pregnant or after you have given birth? Please explain. Please explain. (Remind them to consider social discrimination, privacy disclosure, expense issues, time issues, etc.).
   3. What would be an acceptable time to spend in the clinic for screening?
   4. What would make universal screening more acceptable (Remind them: for the women and for themselves)?

**Section B. How often to Screen**

**Guidelines for universal screening for perinatal depression are inconsistent with respect to how often during pregnancy and after birth women should be screened for depression. The American College of Obstetricians and Gynecologists (ACOG) and the National Perinatal Depression Initiative (NPDI) firmly recommends screening of women at least once during pregnancy and after birth using standardized validated tools. Antenatal depression rates in six counties/districts in six provinces in China determined the antenatal depression rate is 14% in first trimester, 13% in second trimester, and 11% in third trimester. Furthermore, the course of depression is U-shaped, higher in first and third trimester but these findings can be misleading given low number of studies examining depression in first trimester and lack of utilization of antenatal care among women with mental health problems.**

1. How frequently should women be screened for depression during pregnancy and why?
2. How frequently should women be screened for depression after pregnancy and why?
3. What is the frequency of screening that would be acceptable to you?
4. If you thought you were developing emotional distress what would you do? (Reminder: ask if they would consider: nothing, engage in self-care, talk to a friend or family members, see a traditional doctor, see a family doctor, and see a doctor in a psychiatric hospital).

**Section C. Referral Pathways for Management of Perinatal Depression**

**Internet-based Cognitive Behavior Therapy. Cognitive behavior therapy includes a problem solving approach that enables women to change ways of thinking by becoming aware of negative interpretations (eg, thoughts, beliefs, and attitudes) and behaviors that perpetuate these negative interpretations. To deliver such a program in person is resource intense. In randomized controlled trials internet-based cognitive behavior therapy has been demonstrated to reduce depressive symptoms.**

1. How would you feel about using an internet-based cognitive behavior therapy as a management strategy for emotional distress?
   1. Can you skillfully use smart phones and computers to access the internet? How often do you use your computer and mobile internet?
   2. Do you think women generally are skillful in accessing internet?
   3. What are some potential challenges with women using this approach?
2. What do you think are the advantages of establishing an internet-based management of perinatal depression within primary health care system compared with existing medical approaches?
3. What do you think are the disadvantages of establishing an internet-based management of perinatal depression within primary health care system compared with existing medical approaches?

**Section D. Psychological Support**

**Strong evidence suggests that personal support (eg, psychological counsellor/therapist) combined with internet-based cognitive behavior therapy is more effective in managing perinatal depression. We will be training all health care provider to deliver low-intense psychological support.**

1. What are your thoughts on this approach?
   1. What are potential strengths and challenges?
2. Do you feel policy decision makers, healthcare providers, women, and families will like this approach? Why?
3. Should this psychological support be available through internet?

**Overall Impression**

1. What are your views on implementing a perinatal depression screening and management strategy in the primary healthcare system in [name of the city]?
   1. Do you see yourself as an advocate for the program?
