# Supplementary material for: What stakeholders think: perceptions of perinatal depression and screening in China’s primary care system
Source: BMC Pregnancy Childbirth. 2021 Jan 6;21:15. doi: 10.1186/s12884-020-03473-y (PMC7789622; doi:10.1186/s12884-020-03473-y)
Supplement: Supplementary file 1 — Additional file 1. Interview Guide for Families. Interview Guide for Healthcare Provider. Interview Guide for Women. Interview Guide for Policymaker. [file 12884_2020_3473_MOESM1_ESM.zip › BMC Premji et al File 4_Guide Interview_Policy D-MR4.docx]

**Interview Guide for Policy Decision Maker**

Review Information Sheet and Consent Form and secure signatures.

Thank you for agreeing to participate in this interview and having this interview recorded. As explained, the interview will take approximately 60-90 minutes and will take the format of question and answers. If the question involves state secrets, you can also refuse to answer. The more detailed your answers to relevant questions, the better. At the end you will be given an opportunity to share anything that else that we have not covered. Thank you for your cooperation. Do you have any questions? If not, we are going to start the interview.

Before we begin, I am going to start recording and request that you indicate you give your permission to record this interview. You do not need to state your name or any other details that may identify you. Thank you!

**Section A. Universal Screening**

**Currently there is fair to moderate evidence that universal screening for depression should be integrated into primary care.**

1. Is this evidence currently applied in healthcare system(s) in [name of city example Ma’anshan city]?

**If yes,** how is it applied?

- 1. Does the primary healthcare system provide universal screening for depression for women during pregnancy and after they give birth (perinatal period)?
  2. Do you think the current primary health care system pays enough attention to perinatal depression? If no, why do you think that is the case? How can the primary healthcare system be improved to pay enough attention to the issue of perinatal depression?
  3. What is the main way women are identified with perinatal depression?
  4. In what setting or institution is perinatal depression first diagnosed and treated?

**If no,** Do you think it is necessary to provide these services? What is the basis?

**If no,** why do you think it is not applied?

1. What are the difficulties in integrating this evidence into policy?
2. What are the difficulties in integrating this evidence into practice?
3. What are the difficulties in the primary health care system that makes it difficult to integrate this evidence?
4. What challenges do you anticipate if our team integrates universal screening for perinatal depression in primary healthcare system?
5. In [name of city example Ma’anshan city], what policy and institutional support can be gained by our research team to integrate universal screening of perinatal depression within primary healthcare system?

**Section B. How often to Screen**

**Guidelines for universal screening for perinatal depression are inconsistent with respect to how often during pregnancy and after birth women should be screened for depression. The American College of Obstetricians and Gynecologists (ACOG) and the National Perinatal Depression Initiative (NPDI) firmly recommends screening of women at least once during pregnancy and after birth using standardized validated tools. Antenatal depression rates in six counties/districts in six provinces in China determined the antenatal depression rate is 14% in first trimester, 13% in second trimester, and 11% in third trimester. Furthermore, the course of depression is U-shaped, higher in first and third trimester but these findings can be misleading given low number of studies examining depression in first trimester and lack of utilization of antenatal care among women with mental health problems.**

1. How frequently should women be screened for depression during pregnancy and why?
2. How frequently should women be screened for depression after pregnancy and why?

**Section C. Referral Pathways for Management of Perinatal Depression**

**Internet-based Cognitive Behavior Therapy. Cognitive behavior therapy includes a problem solving approach that enables women to change ways of thinking by becoming aware of negative interpretations (eg, thoughts, beliefs, and attitudes) and behaviors that perpetuate these negative interpretations. To deliver such a program in person is resource intense. In randomized controlled trials internet-based cognitive behavior therapy has been demonstrated to reduce depressive symptoms.**

1. What are your perspective on delivering cognitive behavior therapy by internet to women with depression during pregnancy and postpartum?
   1. What some potential challenges with the uptake of this approach?
   2. What do you think are the advantages of establishing an internet-based management of perinatal depression within primary health care system compared with existing medical approaches?
   3. What do you think are the disadvantages of establishing an internet-based management of perinatal depression within primary health care system compared with existing medical approaches?
2. At present, in the primary healthcare system in Ma 'anshan city, how are severe cases of perinatal depression referred (Referral pathway)?
3. Is the primary healthcare system tracking, registering, summarizing and reporting on her diagnosis and treatment? If so, what aspects are recorded? Is this an important issue?
4. Integrating internet-based cognitive behavior therapy within the primary healthcare system is expensive.
   1. What policy may support reimbursement of cost of this therapy after the study?
   2. Do you have any suggestions about how to manage issues related to cost and payment, such as the cost of screening, and the internet-based cognitive behavior therapy program?
   3. Who (eg, corporations, insurance companies) do you think would be willing to cover or subsidize the cost of this therapy?

**Section D. Psychological Support**

**Strong evidence suggests that personal support (eg, psychological counsellor/therapist) combined with internet-based cognitive behavior therapy is more effective in managing perinatal depression. We will be training all health care provider to deliver low-intense psychological support.**

1. What are your thoughts on this approach?
   1. What are potential strengths and challenges?
2. Do you feel policy decision makers, healthcare providers, women, and families will like this approach? Why?

**Overall Impression**

1. What are your views on implementing a perinatal depression screening and management strategy in [name of city example Ma’anshan city]?
   1. Please comment on feasibility, effectiveness, accessibility, and acceptability of the program to policy decision makers, healthcare providers, women, and families.
   2. What are potential challenges that we will need to overcome?
   3. How can we overcome these?
   4. Do you see yourself as an advocate for the program?
